# Change in nutritional status among women of childbearing age in India (1998–2016)

**DOI:** 10.1002/osp4.433

**Published:** 2020-06-12

**Authors:** Sanni Yaya, Bishwajit Ghose

**Affiliations:** ^1^ Faculté de Médecine Université de Parakou Parakou Benin; ^2^ Institute of Nutrition and Food Science University of Dhaka Dhaka Bangladesh

**Keywords:** India, National Family Health Survey, nutritional status, women

## Abstract

**Introduction:**

In absolute numbers, India has more undernourished people than all the countries in sub‐Saharan Africa combined. In parallel with the high rates of hunger and undernutrition, the country has been undergoing rapid demographic and dietary transition marked by an increased prevalence of overweight/obesity, particularly among women.

**Objective:**

To measure the changing prevalence of overnutrition during last two decades, as well as to identify the associated sociodemographic correlates among pregnant and non‐pregnant women in India.

**Methods:**

This was a cross‐sectional study based on data from the latest round of National Family Health Survey (2015–2016) conducted among urban and rural women. Participants were 687,876 women (655,850 non‐pregnant and 32,026 pregnant) aged between 15 and 49 years. Nutritional status was assessed in terms of body mass index (BMI) using the cut‐off for Asian population.

**Results:**

Since 1998–1999, the prevalence of underweight has decreased by 9.2%, while that of overweight (BMI = 23–27.4 kg/m^2^) and obesity (BMI ≥ 27.5 kg/m^2^) has increased by 6.7% and 3.4%, respectively. Results of multivariable regression analysis revealed significant association between nutritional status and age, parity residency, educational level, religious affiliation, household wealth quintile, and TV watching behaviour. Of those, age and wealth status appeared to be the strongest predictors among both pregnant and non‐pregnant women.

**Conclusion:**

Since 1998, there has been a considerable drop in the prevalence of underweight and rise in the prevalence of overweight and obesity. Significant sociodemographic variations exist in nutritional status, notably age and financial situation, which should be highlighted in national nutrition policymaking and intervention programmes.

## 1 BACKGROUND

Maintaining adequate nutrition is central to good health outcomes and quality of life at individual level and a healthy workforce and sustainable socioeconomic development at national level.^1^, ^2^ No other aspect of life has as pervasive an impact on physical, psychosocial and overall well‐being as does nutrition. As promoting nutritional status is embodied as a key prerequisite for achieving the Millennium and Sustainable Development Goals of the United Nations, addressing global hunger and undernutrition remains a key priority of national governments and international donors.^3^, ^4^ Considerable progress has been made by many countries in terms of reducing undernutrition since the introduction of MDGs in 1990. At the same time, overnutrition has gradually emerged to be an equally important public health concern especially in the countries traditionally characterized by high rates of undernutrition, for example, countries in sub‐Saharan Africa and South Asia. According to WHO estimates, globally about 1.9 billion adults are now affected by excess nutrition, while 462 million are underweight.^5^

Growing body of evidence from the medical literature suggests that overnutrition is no longer a phenomenon unique to the affluent economies, as the burden of overweight and obesity is reaching epidemic proportions especially in the fast developing countries such as China, India and Bangladesh.^6^, ^7^, ^8^, ^9^ Commonly recognized as a global hotspot for maternal and child undernutrition, India now accounts for the highest number of overweight and fifth highest number of people with obesity in the world.^10^ In India, the prevalence for adult population living with excess body weight has been increasing steadily during last two decades, with the prevalence being particularly higher among women compared with men.^11^ Being a major risk factor of non‐communicable chronic diseases (NCDs), the increasing prevalence of overweight and obesity has duly translated to a dramatic increase in the burden of diseases such as diabetes, hypertension, cardiovascular diseases, to name a few.^7^, ^8^ While the rate of undernutrition has been declining at the same time, the changing epidemiological landscape with higher burden of overnutrition and NCDs is posing significant challenges for population health and healthcare system in the country.^12^, ^13^

Obesity is a multifaceted problem whose risk factors vary across and within countries and having wide‐ranging medical and socioeconomic consequences.^12^, ^13^, ^14^ There has been a growing volume of epidemiological studies exploring the root causes overnutrition in India.^15^, ^16^, ^17^ The findings indicate that the shift from undernutrition to overnutrition is mostly attributable to the demographic transition involving population ageing, rapid urbanization and socioeconomic transition that is triggering changes in lifestyle behaviour and dietary patterns. This epidemiological shift, also known as nutrition transition, is a phenomenon that involves the coexistence of undernutrition and overnutrition, which is commonly referred to as the double burden of malnutrition.^6^, ^18^, ^19^

From the perspective of public health situation in India, addressing the challenges posed by double burden of malnutrition is particularly difficult due to the complex demographic distribution of the problem. For example, the occurrence of obesity in mothers with stunted child in the same family requires an integrated household based rather than individual‐based approach to nutritional interventions for the double burden.^20^ From this view, tackling obesity among women is a key public health priority for countries like India as maternal obesity that is associated with a host of pregnancy and child health issues (e.g., preterm birth, low birthweight) with potential impacts on child's nutritional status in the later stages of life.^21^ Addressing maternal obesity is facilitated by effective health policymaking based on the evidences from sociodemographic analysis of the burden of distribution of the issue. Based on this understanding, this study was conducted to provide an update on the prevalence of overnutrition among married women in both rural and urban areas of India. Data were nationally representative and collected from three rounds of National Family Health Survey (NFHS) conducted between 1998 and 2016 to calculate the changing pattern of nutritional status in terms of body mass index (BMI). Additionally, the latest survey (2015–2016) was used to assess the sociodemographic predictors of BMI.

## 2 METHODS

### 2.1 Data source and survey methods

This study was based on data from NFHS conducted in India between 1998 and 2016. NFHS started in the early 1990s and has become a crucial source of data on population, health and nutrition in the country. Participants are adult women (15–49 years), men (15–54 years) and young children (≤59 months). NFHS are conducted under the stewardship of the Ministry of Health and Family Welfare (MoHFW) of Government of India and is a collaborative effort of a large number of organizations including the International Institute for Population Sciences (IIPS). For sampling design, NFHS employs a stratified two‐stage technique. The first stage involves selection of clusters in urban and rural areas called primary sampling units (PSUs) containing a certain number of households (~30). The second stage involves systematic selection of 22 households from each cluster. Funding for the surveys were provided by MoHFW as well as by some international agencies such as United States Agency for International Development (USAID), Department for International Development (DFID), the Bill and Melinda Gates Foundation, UNICEF, UNFPA and the MacArthur Foundation, with technical assistance by ICF international USA. Detailed versions of sampling procedure are available through the NFHS reports^22^ and previous publications.^15^, ^17^, ^23^

### 2.2 Study variables

The outcome variable for this study was nutritional status measured in terms of BMI. The outcome variable was overweight/obesity status, which generally results from excessive intake of nutrients, generating an energy imbalance between food consumption and energy expenditure.^24^ NFHS collects anthropometric information, for example, height and weight for a subsample of households only. Weight was measured using Seca 874 digital scale and height by Seca 213 stadiometer. BMI was calculated by using the standard formula (weight in kg/height in m^2^) and was classified according to cut‐off based on risk of type 2 diabetes and cardiovascular disease for Asian population: <18.5 kg/m^2^ (underweight), 18.5–22.9 kg/m^2^ (acceptable risk), 23–27.4 kg/m^2^ (increased risk) and >27.5 kg/m^2^ (high risk).^12^ The terms ‘overweight’ and ‘increased risk BMI’ and ‘obesity’ and ‘higher risk BMI’ were used synonymously in the text.

Independent variables were selected based on their demonstrated/theoretical association with body weight status and their availability on the dataset. The following were finally included in the analysis: age (15–19/20–24/25–29/30–34/35–39/40–44/45–49); parity (nulliparous/ primiparous/multiparous); currently pregnant (yes/no); education (no education/primary/secondary/higher); residence type (urban/rural); wealth quintile (poorest/poorer/middle/richer/richest); religion (Hinduism/other); frequency of TV watching (never, less than once a week/at least once a week/almost every day). For the calculation household wealth status, instead of direct income the volume of durable goods (e.g., TV, radio and bicycle) possessed by the household as well as and housing quality (e.g., type of floor, wall and roof) are taken into consideration. Each item is assigned a factor score generated through principal component analysis (PCA), which are then summed and standardized for the households.^25^ These standardized scores place the households in a continuous scale based on relative wealth scores. The scores thus obtained from a continuous scale and subsequently categorized into quintiles to rank the household as poorest/poorer/middle/richer/richest to richest.^13^

### 2.3 Data analysis

Data were analysed using Stata version 14. As NFHS employs cluster sampling techniques, ‘svy’ command was used to account for the survey design. Using *χ*² tests, the sociodemographic characteristics of the sample population across the BMI categories were presented as percentages. The prevalence rates of overweight/obesity (23–27.4 and >27.5 kg/m^2^) were shown as bar charts.

Second, multivariable logistic regression analyses were performed to identify the variables significantly associated with BMI status. Regression models were further was stratified into subpopulation groups by pregnancy status, as pregnant women are less likely to have overweight/obesity compared with non‐pregnant women. The level of significance was set at *p* < 0.05 for all analyses. After the multivariate analysis, variance inflation factor (VIF) test was performed to check for multicollinearity between independent variables has been checked. VIF values ranged from 1.01 to 2.01, denoting the absence of any multicollinearity.

Relative contribution of the explanatory variables to BMI status were also reported. This was performed as a regression postestimation procedure in Stata that calculates the individual contribution of a variable divided by the sum of the total contribution of all variables. This represents the proportional contribution of a variable in terms of total variance explained in the outcome variables. This was considered necessary as the odds ratios from regression analysis do not reflect the relative importance or weight of explanatory variables. It is however, important to note that this procedure does not reflect any unexplained variance.

## 3 RESULTS

### 3.1 Descriptive analysis

Sociodemographic profile of the sample population was shown in Table 1. A greater percentage of women who had underweight were in the lower age groups, whereas, those who had overweight and obesity were in the higher age groups. The percentage of overweight/obesity was also higher among women with more than two children, non‐pregnant, had secondary level education, rural resident (for overweight), from the households with higher (richer/richest) wealth status, followers of Hinduism, and watched TV almost everyday.

**TABLE 1:** Distribution of BMI categories over the sample population (2015–2016)

Variables	Underweight	Normal weight	Increased risk (overweight)	Higher risk (obesity)	*p* value
Individual factors					
**Age**					<0.001
15–19 (*n* = 122,416)	31.7	19.2	6.0	3.3	
20–24 (*n* = 120,859)	21.5	20.5	12.6	7.6	
25–29 (*n* = 113,202)	15.0	17.6	17.1	13.7	
30–34 (*n* = 95,473)	10.3	13.2	17.0	17.4	
35–39 (*n* = 89,044)	8.3	11.7	17.1	19.6	
40–44 (*n* = 75,416)	7.0	9.2	15.3	19.4	
45–49 (*n* = 71,466)	6.3	8.6	15.0	19.0	
**Parity**					<0.001
Nulliparous (*n* = 218,010)	44.6	33.6	17.7	12.6	
Primiparous (*n* = 92,028)	11.7	14.2	16.0	15.3	
Multiparous (*n* = 377,838)	43.7	52.3	66.3	72.1	
**Currently pregnant**					<0.001
No (*n* = 655,850)	97.2	94.5	95.2	96.9	
Yes (*n* = 32,026)	2.8	5.5	4.8	3.1	
**Education**					<0.001
No education (*n* = 193,773)	29.9	29.2	25.4	20.5	
Primary (*n* = 87,148)	12.1	12.5	13.0	12.9	
Secondary (*n* = 329,351)	49.3	46.1	46.4	50.5	
Higher (*n* = 77,604)	8.7	12.2	15.3	16.1	
Household/community level factors					
**Residence type**					<0.001
Urban (*n* = 199,244)	23.2	29.5	43.1	43.3	
Rural (*n* = 488,632)	76.8	70.5	56.9	56.7	
**Wealth quintile**					<0.001
Poorest (*n* = 131,548)	27.7	20.9	9.0	3.8	
Poorer (*n* = 147,658)	25.3	21.9	15.1	8.8	
Middle (*n* = 145,133)	20.8	21.2	21.1	16.9	
Richer (*n* = 136,001)	15.8	19.2	26.0	30.0	
Richest (*n* = 127,536)	10.5	16.8	28.8	40.5	
**Religion**					<0.001
Hindu (*n* = 510,755)	83.1	81.2	79.0	75.8	
Muslim (*n* = 177,121)	16.9	18.8	21.0	24.2	
**TV watching**					
Never (*n* = 168,150)	30.8	25.9	15.5	9.9	22.9
>once/week (*n* = 52,068)	7.8	6.8	4.8	3.6	6.2
At least once/week (*n* = 80,501)	11.1	10.3	9.0	7.7	9.9
Almost every day (*n* = 387,157)	50.3	57.0	70.7	78.9	60.9

**FIGURE 1:**
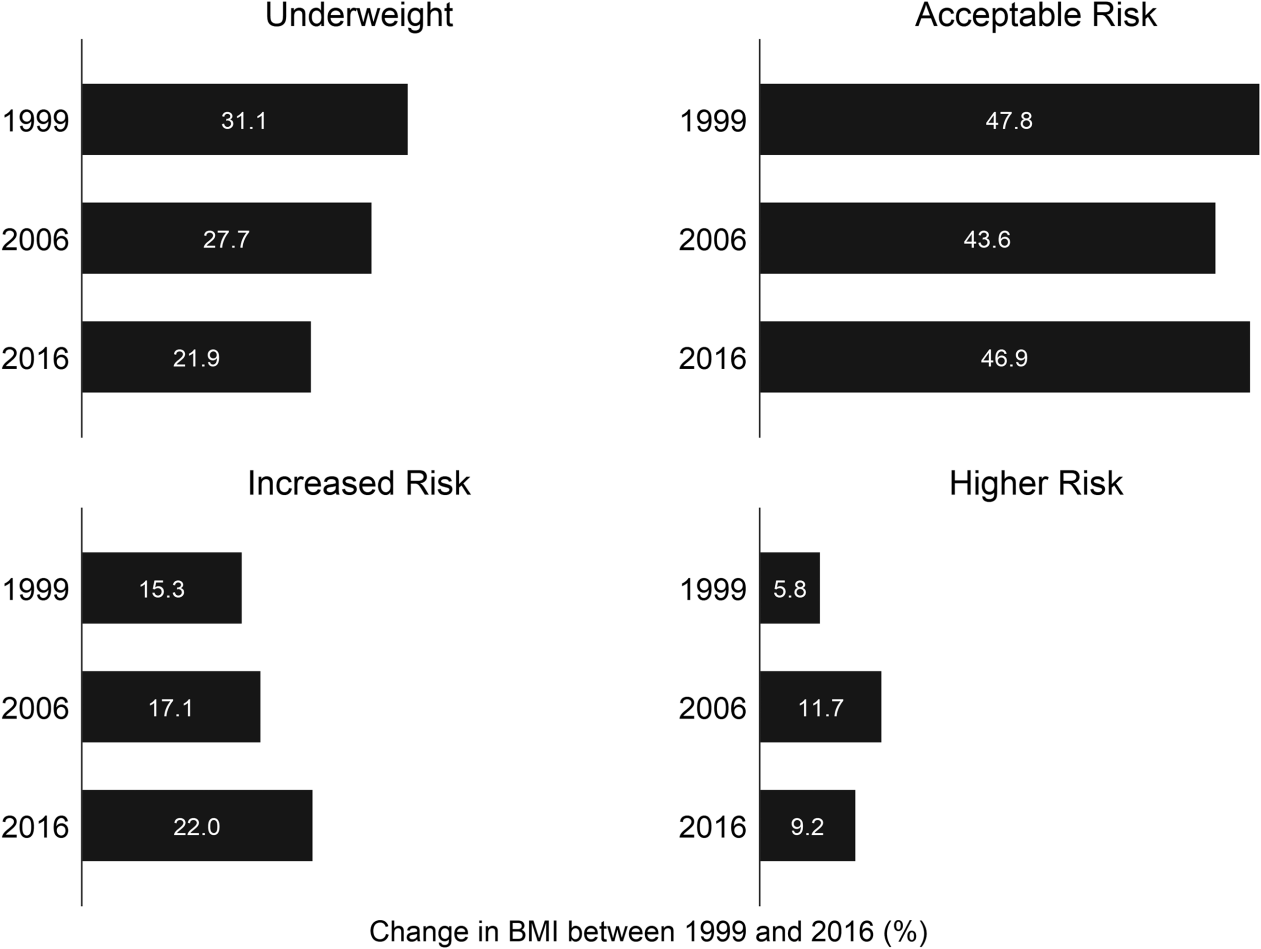
Change in BMI categories between 1999 and 2016 among Indian women There has been a net decline in the prevalence of underweight women: 31.1% in 1999 versus 21.9% in 2016 between 1999 and 2016. In 1999, the prevalence of women living with BMI of increased risk (23–27.4 kg/m^2^) and higher risk (>27.5 kg/m^2^) were, respectively, 15.3% and 5.8%, compared with, respectively, 22.0% and 9.2% in 2015–2016

### 3.2 Factors associated with overweight and obesity

Results of multivariable regression analysis showed significant association between BMI with age, residency, educational level, religious affiliation and wealth index among both non‐pregnant and pregnant women (Table 2).

**TABLE 2:** Factors associated with BMI status among non‐pregnant women in India (2015–2016)

	Pregnant sample			Non‐pregnant sample		
	Underweight	Increased risk/overweight	Higher risk/obese	Underweight	Increased risk/overweight	Higher risk/obese
**Age groups (15–19)**	1.980^***^ [1.763,2.224]	0.178^***^ [0.161,0.197]	0.0722^***^ [0.0632,0.0824]	2.267^***^ [2.013,2.553]	0.205^***^ [0.181,0.231]	0.0920^***^ [0.0733,0.115]
20–24	1.292^***^ [1.149,1.452]	0.306^***^ [0.278,0.338]	0.138^***^ [0.121,0.157]	1.933^***^ [1.741,2.146]	0.312^***^ [0.289,0.336]	0.163^***^ [0.148,0.181]
25–29	0.909 [0.805,1.026]	0.521^***^ [0.471,0.576]	0.285^***^ [0.249,0.326]	1.399^***^ [1.257,1.558]	0.484^***^ [0.449,0.522]	0.320^***^ [0.291,0.352]
30–34	0.867^*^ [0.758,0.992]	0.704^***^ [0.631,0.786]	0.541^***^ [0.469,0.625]	1.149^*^ [1.021,1.293]	0.682^***^ [0.629,0.739]	0.606^***^ [0.548,0.670]
35–39	0.979 [0.847,1.132]	0.828^**^ [0.736,0.933]	0.716^***^ [0.614,0.836]	1.090 [0.960,1.238]	0.759^***^ [0.696,0.828]	0.726^***^ [0.652,0.808]
40–44	1.007 [0.861,1.177]	0.862^*^ [0.758,0.981]	0.783^**^ [0.663,0.925]	1.089 [0.949,1.250]	0.918 [0.836,1.008]	1.028 [0.918,1.151]
45–49	1	1	1	1	1	1
**Parity (nulliparous)**	1	1	1	1	1	1
Primiparous	0.870^***^ [0.850,0.892]	1.258^***^ [1.226,1.290]	1.450^***^ [1.394,1.508]	1.331^***^ [1.220,1.451]	0.976 [0.911,1.046]	1.186^**^ [1.057,1.331]
Multiparous	0.946^***^ [0.925,0.968]	1.228^***^ [1.199,1.258]	1.481^***^ [1.429,1.536]	1.298^***^ [1.169,1.441]	0.879^**^ [0.809,0.956]	0.954 [0.826,1.102]
**Education (none)**	1	1	1	1	1	1
Primary	0.995 [0.948,1.044]	1.103^**^ [1.030,1.180]	1.229^***^ [1.088,1.387]	0.907^**^ [0.851,0.966]	1.067 [0.995,1.145]	1.249^***^ [1.120,1.392]
Secondary	0.963^*^ [0.928,0.999]	1.099^***^ [1.043,1.158]	1.201^***^ [1.093,1.320]	0.841^***^ [0.799,0.884]	1.244^***^ [1.178,1.314]	1.380^***^ [1.266,1.504]
Higher	0.855^***^ [0.817,0.894]	1.068^*^ [1.008,1.133]	0.978 [0.882,1.085]	0.660^***^[0.611,0.714]	1.331^***^ [1.244,1.424]	1.292^***^ [1.169,1.428]
**Residence (urban)**	1	1	1	1	1	1
Rural	0.965^**^ [0.941,0.989]	0.844^***^[0.819,0.871]	0.690^***^ [0.655,0.727]	1.016 [0.968,1.066]	0.861^***^ [0.828,0.895]	0.737^***^[0.699,0.777]
**Wealth quintile (lowest/poorest)**	1	1	1	1	1	1
Poorer	0.939^***^ [0.910,0.969]	1.424^***^ [1.347,1.506]	1.903^***^ [1.667,2.172]	0.893^***^ [0.845,0.944]	1.422^***^ [1.321,1.530]	2.094^***^ [1.780,2.463]
Middle	0.895^***^ [0.865,0.926]	1.823^***^ [1.722,1.930]	2.905^***^ [2.549,3.310]	0.757^***^ [0.711,0.806]	1.898^***^ [1.761,2.047]	3.601^***^ [3.071,4.222]
Richer	0.848^***^ [0.816,0.881]	2.042^***^ [1.923,2.169]	4.396^***^ [3.851,5.019]	0.679^***^ [0.632,0.729]	2.323^***^ [2.147,2.513]	5.691^***^ [4.848,6.682]
Highest/richest	0.713^***^ [0.683,0.743]	2.344^***^ [2.200,2.497]	5.814^***^ [5.075,6.660]	0.537^***^ [0.494,0.584]	2.584^***^ [2.377,2.809]	7.852^***^ [6.663,9.252]
**Religion (Hindu)**	1	1	1	1	1	1
Others	0.690^***^ [0.674,0.706]	1.210^***^ [1.176,1.244]	1.064^*^ [1.012,1.117]	0.683^***^ [0.653,0.715]	1.162^***^ [1.118,1.208]	1.216^***^ [1.152,1.284]
**TV watching (never)**	1	1	1	1	1	1
>Once/week	0.946^**^ [0.908,0.985]	1.001 [0.939,1.068]	0.898 [0.788,1.023]	0.985 [0.916,1.059]	1.013 [0.930,1.103]	1.057 [0.911,1.227]
At least once/week	0.948^**^ [0.914,0.983]	1.035 [0.979,1.093]	0.893^*^ [0.801,0.995]	0.955 [0.895,1.020]	1.091^*^ [1.016,1.172]	1.080 [0.956,1.220]
Almost every day	0.981 [0.952,1.011]	1.119^***^ [1.069,1.171]	1.099^*^ [1.006,1.200]	0.931^**^ [0.882,0.982]	1.235^***^ [1.164,1.310]	1.407^***^ [1.272,1.556]

*Note.* N.B. number represents odds ratios with 95% confidence intervals. Reference category for BMI is *acceptable risk.*

* *p* < 0.05,

** *p* < 0.01,

*** *p* < 0.001.

For non‐pregnant women, compared with these in the highest age group (45–49 years), those in the lower age groups had significantly lower odds of having overweight and obesity. Compared with women with no children, those with one or more had higher odds of having overweight and obesity. Having secondary (odds ratio = 0.963, 95% confidence interval [CI] = 0.928, 0.999) and higher [odds ratio = 0.855, 95% CI = 0.817, 0.894] education showed protective effect against being underweight. Educational status was only marginally associated with overweight but relatively stronger association with having obesity: odds ratio of 1.229 [95% CI = 1.088, 1.387] for primary and 1.201 [95% CI = 1.093, 1.320] secondary education. Rural residence showed a protective effect for overweight [odds ratio = 0.844, 95% CI = 0.819, 0.871] and obesity [odds ratio = 0.690, 95% CI = 0.655, 0.727]. Household wealth status showed a strong negative association with underweight and positive association with overweight/obesity. For instance, in comparison with the poorest ones, women in the household of highest wealth quintile had the lowest odds of being underweight [odds ratio = 0.713, 95% CI = 0.683, 0.743] and highest odds of having overweight [odds ratio = 2.344, 95% CI = 2.200, 2.497] and obesity [odds ratio = 5.814, 95% CI = 5.075, 6.660]. The odds of overweight and obesity increased progressively with higher wealth quintiles, while for underweight the odds decreased as wealth quintile increased. Those belonging to religious groups other than Hinduism had lower odds of being underweight [odds ratio = 0.690, 95% CI = 0.674, 0.706] but lower odds of having overweight [odds ratio = 1.210, 95% CI = 1.176, 1.244] and obesity [odds ratio = 1.064, 95% CI = 1.012, 1.117]. Frequency of TV watching showed an inverse association with body weight such that most frequent watching (almost everyday) increased the odds of overweight [odds ratio = 1.119, 95% CI = 1.069, 1.171] and, albeit marginally, for obesity [odds ratio = 1.099, 95% CI = 1.006, 1.200].

The pattern of associations observed for pregnant women were similar to those in the non‐pregnant sample except for differences in terms of parity. As opposed to the non‐pregnant sample, the odds of underweight were higher among primiparous [odds ratio = 1.331, 95% CI = 1.220, 1.451] and multiparous women [odds ratio = 1.298, 95% CI = 1.169, 1.441]. The positive association between TV watching and having overweight [odds ratio = 1.235, 95% CI = 1.164, 1.310] and obesity [odds ratio = 1.407, 95% CI = 1.272, 1.556] was more pronounced among the pregnant sample.

Results of relative contribution analysis (Table 3) show that age was most significant predictor of BMI in the pooled sample followed by wealth quintile, parity, TV watching and place of residence. After stratifying by current pregnancy status, age remained the most important predictor of BMI in the non‐pregnant sample, whereas for pregnant sample wealth quintile stood out as the most important predictor.

**TABLE 3:** Relative contribution of the explanatory factors to nutritional (BMI) status

Variables	Percent contribution		
	Overall	Non‐pregnant	Pregnant
Age	34.63	35.05	26.28
Wealth quintile	28.08	28.01	34.03
Parity	14.62	15.58	1.74
TV watching	7.26	7.27	9.75
Residency	7.15	7.15	9.30
Education	4.29	4.25	11.61
Religion	2.77	2.70	7.29
Pregnancy	1.19	‐	‐

## 4 DISCUSSION

The findings suggest a slow but steady progress in reducing the prevalence of undernutrition, as well as a steady and significant rise in the prevalence of overweight/obesity at the same time. The prevalence of undernutrition dropped by about one‐tenth between 1998–1999 and 2015–2016. As of 2015–2016, more than one‐fifth and almost one‐tenth women were living with overweight and obesity, respectively, compared with about one‐tenth and less than one‐tenth in 1998–1999. The change in BMI across the survey years was statistically significant (*p* < 0.05). The prevalence of being in the underweight category was higher among women in the lower age groups, whereas, that of increased and higher risk was higher among women in the higher age groups, reflecting a clear age gradient in nutritional status. Increasing age is known predictor of gaining excess body weight, which requires special age‐specific intervention techniques.^26^ Obesity is not only a risk factor for developing metabolic syndrome (cluster of conditions that occur together, e.g., cardiovascular diseases and type 2 diabetes) but also of maternal and neonatal morbidity mortality, a matter of concern for public health in India. With the growing burden of overweight/obesity, developing age appropriate obesity control and intervention programmes should be prioritized.

In line with previous findings, women with higher parity were found to be more likely to have overweight and obesity and less likely to have underweight, implying a beneficial effect of motherhood against underweight.^27^, ^28^, ^29^ This finding also underscores the importance of regulating excess body weight among women with higher parity. As women tend to accumulate body fat at each pregnancy, higher parity can heighten the risk of developing moderate to extreme obesity and associated complications. Interestingly, the association between parity was reversed among pregnant women. However, pregnancy itself is a strong predictor of overweight/obesity, which can confound the association between parity and body weight. The nature of this conflicting relationship between parity and body weight among pregnant women could have been better understood provided data on prepregnancy body weight were available. Future studies should focus on exploring the relationship by comparing prepregnancy and postpregnancy body weight.

Women's socioeconomic status, such as educational and wealth situation showed a positive association with body weight, with wealth status having a more pronounced effect. Women with relatively higher education had lower odds of being underweight and higher odds of having overweight and obesity than those with no education. The positive effect of education on nutritional status may reflect the beneficial role that education plays on one's financial well‐being. As the analysis further reveals, wealth category was the most notable predictor of nutritional status among both pregnant and non‐pregnant women. There was a dose–response relationship between the two, with higher wealth category significantly reducing the odds of undernutrition and increasing that of overweight and obesity. Better education and financial status are generally associated with more sedentary type of occupation and lifestyle, which exerts a cumulative effect on excess body weight gain. Lower caloric expenditure and higher consumption of energy dense food are common characteristics of wealthy families and are reported to be a major contributor the expansion of the obesity epidemic in the developing countries. Apart from that, religion also appeared to be an important contributing factor to overnutrition as women other than Hinduism. The underlying causes behind this may very well be those related to dietary/nutritional practices and perception of nutritional status/body weight.

The role of sedentary lifestyle on overnutrition is a well‐established one, as is supported by the finding that women who reported watching TV almost everyday had higher percentages of having overweight and obesity. However, in the adjusted multivariate analysis, TV watching did not show any significant association with overweight or obesity. The association between age, residence, religion, education and wealth status remained significant even after stratifying by parity. Across the same wealth and education categories, women with one/more than one child had comparatively higher odds of having overweight and obesity than those with no children. For instance, the odds of being overweight among primiparous/multiparous women were higher among both in the lowest (1.536 vs. 1.380) and highest (11.055 vs. 4.004) wealth quintile.

The findings on the association between overnutrition and age,^30^, ^31^ residency,^16^ education,^31^, ^32^ religion^33^ and wealth status^31^, ^32^, ^33^ are in line with previous studies. Literature review reveals varying degrees of prevalence rates of overweight and obesity, ranging from a combined prevalence of 42.3% at subnational level to 29.9% at national level (NFHD 2005–06).^26^ As per the analysis of a review article, the combined prevalence among ever‐married women aged 15–49 years rose from 11% in 1998–1999 (NFHS 2) to 15% in 2005–2006 (NFHS 3).^34^ Another review of NFHS reported that prevalence of obesity increased from 10.6% to 12.6% during the same period.^16^ In comparison, the prevalence of overweight/obesity was slightly higher than in Nepal (27.5%) and lower than in Bangladesh (39.5%).^12^ However, the prevalence rates are not directly comparable as they are likely to differ depending on the cut‐offs values (Asian vs. international cut‐off) and the inclusion/exclusion criteria applied.

India, as an emerging economy and contributor to the advancement of information and technology sector, is undergoing rapid transformation in demographic, epidemiological and sociocultural terms. While the positive influences of these developments on fighting hunger and malnutrition cannot be ignored, underestimating the negative influences on people's nutritional status, for example, rising burden of overnutrition and associated NCDs is likely to incur significant costs in the form of higher healthcare expenditure and productivity loss. Hence, from population health perspective, it is of utmost importance to prioritize health and nutrition in public policymaking. Indian subcontinent population is known for their higher susceptibility to several NCDs including cardiovascular diseases^35^ and diabetes,^36^ of which higher than normal BMI is a prominent risk factor. Addressing the rising prevalence of overweight/obesity should therefore be regarded as an urgent public health imperative, which needs to be facilitated by appropriate policy mix and actions by bringing together the relevant stakeholders to ensure an approach that is multidisciplinary and comprehensive in nature and effective in implementation. As a country characterized by stark geographic and sociocultural disparities, applying uniform health and nutrition policies are unlikely produce satisfactory outcomes in terms of addressing the challenges of malnutrition.^37^ More population‐based studies should be carried to explore the culture‐specific factors that will help formulation of more nuanced and locally tailored policies to combat overweight/obesity in the population.

The key strength of this study was the large sample size and the use of nationally representative data which ensures greater robustness of the estimates (prevalence rates). As such, the findings are generalizable for women aged 15–49 years. Data were analysed by complex sampling method, which allows adjusting for the clustered nature of the surveys. Asian cut‐off for BMI values were used instead of the international one, which provides a more precise picture of local situation on overnutrition. Apart from the strengths, there are few important limitations to declare. As the dataset was secondary, the measurement and choice of explanatory variables were limited. Lifestyle behaviour related factors are key determinants of overnutrition,^38^ which are not collected by NFHS. For instance, religion and TV use were used as proxy measures for sociocultural and sedentary behaviour, which are in fact crude indicators and may not reflect the actual effect of the key variables. Moreover, the datasets were cross‐sectional, and hence, the associations imply reciprocity rather than causality or directionality.

## 5 CONCLUSIONS

This was a comprehensive analysis of NFHS data with the objectives of providing an updated scenario on overnutrition among adult non‐pregnant female population in India. Consistent with the existing literature, the findings primarily indicate the co‐occurrence of undernutrition and overnutrition, with undernutrition is predominant among the relatively lower wealth status and overnutrition among the relatively higher wealth status households. Overall, there has been a slow yet steady progress in the prevalence of undernutrition; however, that of overnutrition is on a constant rise especially in the rural areas. The prevalence of overnutrition showed significant sociodemographic variations, which need to be considered in making national nutrition policymaking. Although the data were cross‐sectional and prevent making any causal inferences, the findings provide important insight on the current situation of undernutrition and overnutrition among Indian women. Future researches should emphasize on including sociocultural factors as well as lifestyle and behavioural determinants of overnutrition.

## ETHICAL APPROVAL

All participants gave informed consent prior to taking part in the survey. All NFHS surveys are approved by ICF international as well as an Institutional Review Board (IRB) in respective country to ensure that the protocols follow the US Department of Health and Human Services regulations for the protection of human subjects. Further approval was not necessary as the datasets are available in the public domain in anonymised form.

## CONSENT FOR PUBLICATION

Not applicable.

## AVAILABILITY OF DATASETS

All datasets used in this study are available through the DHS website.

## CONFLICT OF INTEREST STATEMENT

None to declare.
